# Toward Personalized Digital Experiences to Promote Diabetes Self-Management: Mixed Methods Social Computing Approach

**DOI:** 10.2196/60109

**Published:** 2025-01-07

**Authors:** Tavleen Singh, Kirk Roberts, Kayo Fujimoto, Jing Wang, Constance Johnson, Sahiti Myneni

**Affiliations:** 1 McWilliams School of Biomedical Informatics, The University of Texas Health Science Center, Houston, TX, United States; 2 School of Public Health, The University of Texas Health Science Center, Houston, TX, United States; 3 College of Nursing, Florida State University, Tallahassee, FL, United States; 4 Cizik School of Nursing, The University of Texas Health Science Center, Houston, TX, United States

**Keywords:** digital health communities, diabetes self-management, behavior change, affiliation exposure, social networks, deep learning

## Abstract

**Background:**

Type 2 diabetes affects nearly 34.2 million adults and is the seventh leading cause of death in the United States. Digital health communities have emerged as avenues to provide social support to individuals engaging in diabetes self-management (DSM). The analysis of digital peer interactions and social connections can improve our understanding of the factors underlying behavior change, which can inform the development of personalized DSM interventions.

**Objective:**

Our objective is to apply our methodology using a mixed methods approach to (1) characterize the role of context-specific social influence patterns in DSM and (2) derive interventional targets that enhance individual engagement in DSM.

**Methods:**

Using the peer messages from the American Diabetes Association support community for DSM (n=~73,000 peer interactions from 2014 to 2021), (1) a labeled set of peer interactions was generated (n=1501 for the American Diabetes Association) through manual annotation, (2) deep learning models were used to scale the qualitative codes to the entire datasets, (3) the validated model was applied to perform a retrospective analysis, and (4) social network analysis techniques were used to portray large-scale patterns and relationships among the communication dimensions (content and context) embedded in peer interactions.

**Results:**

The affiliation exposure model showed that exposure to community users through sharing interactive communication style speech acts had a positive association with the engagement of community users. Our results also suggest that pre-existing users with type 2 diabetes were more likely to stay engaged in the community when they expressed patient-reported outcomes and progress themes (communication content) using interactive communication style speech acts (communication context). It indicates the potential for targeted social network interventions in the form of structural changes based on the user’s context and content exchanges with peers, which can exert social influence to modify user engagement behaviors.

**Conclusions:**

In this study, we characterize the role of social influence in DSM as observed in large-scale social media datasets. Implications for multicomponent digital interventions are discussed.

## Introduction

Type 2 diabetes (T2D) is responsible for affecting nearly 34.2 million adults, which accounts for 10.5% of the US population [1]. According to a recent report, about US $327 billion was spent on the treatment of diagnosed cases of T2D in the year 2017 alone [1]. In addition to its health and economic burden, T2D also increases the risk of developing other health complications such as heart disease, stroke, kidney failure, and blindness [2]. Modifiable health behaviors such as obesity, physical inactivity, unhealthy eating habits, and tobacco use are major risk factors for developing chronic health conditions such as T2D [2].

Behavior modification is a core component of diabetes self-management (DSM) programs and provides the much-needed support to improve health-related outcomes in individuals with diabetes [3]. It is a complex process, and research has shown that a range of psychological and social processes influence an individual’s engagement in the sustenance of positive health behaviors [4,5]. For example, individuals are more likely to comply with health-related goals and adhere to preventive practices, provided their socially connected peers also engage in similar behaviors by changing their intrapersonal beliefs, attitudes, or knowledge [6,7]. However, the mechanisms underlying such multilevel influences are not fully understood. Such a lack of understanding limits our capabilities to personalize support infrastructure to meet individual needs.

The widespread adoption of digital health technologies, such as mobile apps, wearables, sensors, and digital health communities (DHCs), creates opportunities to design tailored strategies for behavior change [8,9]. These technologies enable in-depth analysis of large-scale individual and population-level trends, providing valuable insights into behaviors, preferences, and social networks. [8,9]. The emergence of various peer-driven health communities has allowed health care consumers to interact with their peers and health care providers to garner social support and gather knowledge on various health-related topics, etc [10-12]. DHCs specific to T2D have been shown to enable their users to seek and receive support and obtain valuable information to improve psychosocial care and health outcomes [13]. These communities provide us with large and invaluable datasets in the form of electronic traces of peer interactions that capture the attitudes and behaviors of large populations in near real time and in natural settings [9]. Analyzing these datasets allows us to understand the individualistic and environmental factors underlying behavior change and develop effective behavior change interventions (BCIs) [14].

Several studies have leveraged peer interactions in DHCs to model human health behavior [15]. Some research studies have explicitly focused on DSM-related DHCs and have analyzed the data generated from these communities to (1) identify the content of peer interactions, such as topics or themes of conversation [16,17], and (2) understand linguistic features of expression among members of DHCs and how that influences social support [18]. However, in a social setting, the content of communication and its context can affect the cognitive state of individuals engaging in a conversation [19,20]. Still, the current research on DSM-related DHCs needs to be more integrative of these components. To develop agile, adaptive, and personalized digital experiences for individuals at risk for T2D or diagnosed with T2D, new approaches are needed that consider multilevel contexts that can influence individual adherence to DSM behaviors. In this paper, we present our methodology using a mixed methods approach that combines qualitative analysis, automated text analysis, and social network analysis (SNA) techniques to characterize the role of context-specific social influence patterns underlying peer-to-peer communication and evaluate how “membership or affiliation” in a specific context is predictive of user engagement in DSM. Such an integrative approach can help us optimize user engagement in digital settings and subsequently leverage these platforms as delivery modalities for DSM.

## Methods

### Ethical Considerations

This study was exempted from human participant ethics review approval by the institutional review board at the University of Texas Health Science Center at Houston (HSC-SBMI-15-0697). We extracted only the messages in the public domain, that is, peer interactions marked public by the community users. To maintain user anonymity, we deidentified the data obtained from the DHC by assigning every community user a unique user identifier. In addition, the researchers had no direct contact with the community users.

### Materials

The American Diabetes Association (ADA) support community is a digital support group for individuals with diabetes (type 1, type 2, or prediabetes) to engage with their peers as well as caregivers [21]. The users of the community interact with one another on a wide variety of topics ranging from medication use, diet, physical activity, and daily monitoring of blood glucose levels. Even though the outcomes among type 1, type 2, or prediabetes are impacted by behaviors (such as lifestyle, medication use, and self-monitoring of blood glucose) that can be heavily influenced by an individual’s social infrastructure, for this research, we focused on interactions related to T2D. The dataset used in this research spanned from 2014 to 2021, consisting of 73,543 messages specific to T2D organized into 7619 unique topics posted by 2374 unique community users. The dataset characteristics across all years are presented in Table 1.

**Figure 1.**
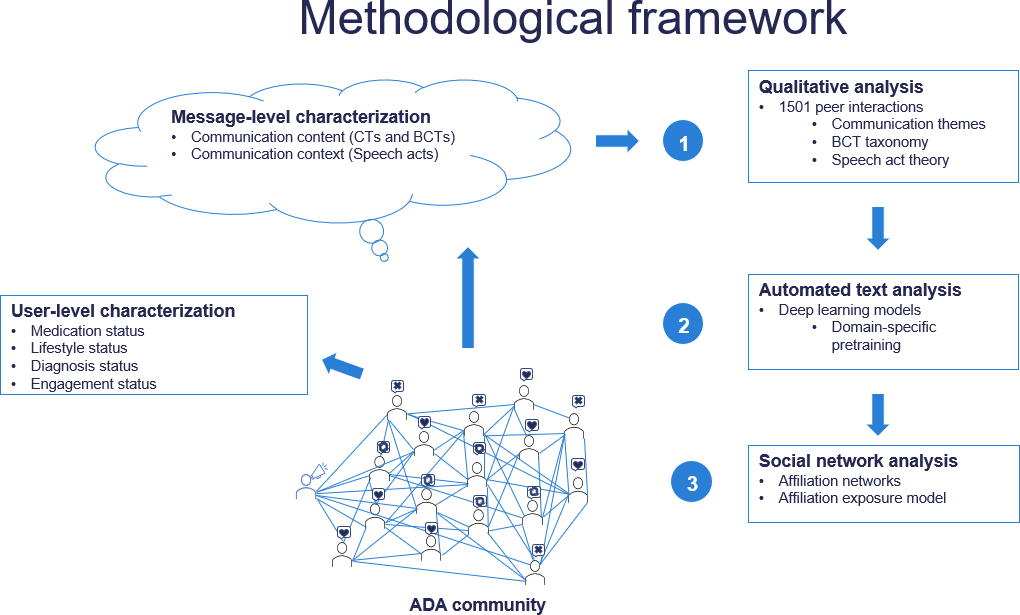
Overall methodological framework. ADA: American Diabetes Association; BCT: behavior change technique; CT: communication theme.

Figure 1 captures the overall methodological framework used in this study and is described in detail below.

**Table 1.** American Diabetes Association dataset characteristics.

	2014	2015	2016	2017	2018	2019	2020	2021
Total messages (n=73,543), n (%)	14,104 (19.2)	18,311 (24.9)	16,859 (22.9)	10,940 (14.9)	6379 (8.7)	3805 (5.2)	2202 (3)	922 (1.3)
Unique topics (n=7619), n (%)	1337 (17.5)	1776 (23.3)	1588 (20.8)	1028 (13.5)	746 (9.8)	587 (7.7)	501 (6.6)	234 (3.1)
Unique users (n=2374), n (%)	597 (25.1)	767 (32.3)	677 (28.5)	458 (19.3)	336 (14.2)	242 (10.2)	206 (8.7)	129 (5.4)

### Characterization of Content and Context Exchanged in Social Ties

#### Qualitative Analysis

The objective of qualitative analysis was to characterize the nature of communication content and underlying context embedded in peer interactions of the ADA community to gain insights into the meaning of peer conversations and the choice of user expressions that affect DSM behaviors. We randomly selected a subset of 1501 forum messages from the original dataset and manually coded them using directed content analysis techniques along the following three dimensions:

1. Communication themes (CTs): Themes capture the essence or meaning of peer conversations and are derived through inductive analysis using grounded theory techniques [22]. These themes provide insights into the theory-driven behavioral constructs prevalent in digital peer interactions.
2. Behavior change techniques (BCTs): For BCTs, we used the BCT taxonomy [23] to identify manifestations of theory-linked BCTs embedded within digital peer interactions. This taxonomy provides a common vocabulary to understand how sociobehavioral and cognitive constructs of existing behavior change theories have been operationalized in BCIs.
3. Speech acts (SAs): To model the communication context underlying digital peer interactions, we used a modified version of Searle’s SA theory [20] to describe how specific content is expressed in human communication using 10 categories of SAs. SA theory can be used to model digital peer interactions to recognize the general attitudes of community users and understand their state of mind by capturing implicit expressions and discourse patterns underlying such peer interactions.

Our qualitative coding schema with definitions of various categories of CTs, BCTs, and SAs can be found in Myneni et al [24] and Singh et al [25].

#### Automated Text Analysis

Given our initial experiments with a conventional multiclass, multilabel classification approach (which yielded poor results) and the inherently imbalanced nature of the dataset (see the Results section), we built a classification approach in which multiple models were combined in a cascading manner [26,27] for classification of the 3 communication attributes (CTs, BCTs, and SAs). We implemented the following deep learning models for performing text classification of peer interactions along the 3 dimensions: recurrent neural networks (RNNs), convolutional neural networks (CNNs), and transformer-based models. The labeled dataset was divided into 3 parts: 80% (1201/1501), 10% (150/1501), and 10% (150/1501) for training, validation, and test sets, respectively. For the implementation of RNNs and CNNs, we used the Adam optimization algorithm to find the best values for each parameter [28]. Specifically, we used the AdamW optimizer to implement the Bidirectional Encoder Representations from Transformers (BERT), set the dropout to 0.1 to avoid overfitting, and used a learning rate of 1 ×10^−5^. We also computed class weights for the loss function to assign a higher weight to the loss encountered by the messages associated with infrequent label categories. To mitigate overfitting and increase the models’ generalization capacity, the validation loss was monitored at every epoch. We found no further decrease in the value of validation loss after 20 epochs for all models that were trained. Therefore, the models were trained for only 20 epochs. We chose model hyperparameters based on their optimal performance on the validation set. We converted the probabilities into label categories based on a threshold value that was calculated using the validation set. RNNs and CNNs were implemented with Keras (developed by Google LLC) [29], and BERT was implemented using PyTorch (developed by Meta Platforms, Inc) [30]. The detailed implementation methodology can be found in Singh et al [31].

### Characterization of Individual Behaviors: Qualitative Analysis

We extracted DSM behavior persona for a subset of users (92 of a total of 205 unique community users) based on their self-reported forum signatures and assigned them behavior profiles based on their DSM strategies [32] and diagnostic features as follows: (1) medication status—whether or not the users take medications; we further classified the medication use to identify oral medicines only (metformin and glipizide) versus injectable only (Novolog and Lantus) versus using both; (2) diagnosis status—newly diagnosed of diabetes (2018 onward) or had pre-existing diabetes (earlier than 2018); and (3) lifestyle profile—whether the users incorporated lifestyle changes (low-carbohydrate or Mediterranean diet, treadmill, and walking) or they did not incorporate any such changes. An example of a self-reported behavior signature is “Diagnosed: February 2017, I went diet controlled with type 2 diabetes. Meds: metformin 500 mg twice a day,” based on which this user was assigned the following behavior persona—medication user, a pre-existing user with T2D, and a user who incorporates lifestyle changes.

### Characterization of Social Ties

#### Overview

Using the labeled peer interactions from the ADA dataset, we characterized the social networks of the 2 DHCs using content-sensitive user-context affiliation networks. These networks consisted of 2 modesT2D the first one being the community users and the second one being the different SA categories. The ties between them recorded the affiliation of each user with each SA in a given CT. The community users were assigned to a specific CT if they had at least exchanged 1 message belonging to the respective CT. For example, in obstacles CT–based social network (Multimedia Appendix 1), the first community user is affiliated with assertion SA, the second community user is affiliated with commissive SA, and the third community user is affiliated with both SAs, given that these users expressed themselves using these categories of SAs in the given CT. We constructed visual representations of various CT-based affiliation networks between community users and SAs. We used Gephi, an open-source network visualization tool, to create and analyze these networks [33].

#### Affiliation Exposure Model

We used 2-mode affiliation networks consisting of 2 distinct sets of nodes—the first set of nodes represents the ADA community users (total n=360‐529, varies by CT), and the second set of nodes represents the various SA categories (*k*=8). We used CT-based social networks, where SAs were further categorized based on community user’s communication styles. The two broad communication styles were as follows: (1) the sender of the message has an intention to “push-in” information to the receiver (using SAs—assertion, stance, declarative, directive, and statement) and (2) “interactive turn-taking,” where the sender might try to engage their peers by pulling out and pushing in information in the form of question, expressive, or emotion. A community user was considered affiliated with a specific SA category only if that user had exchanged a message with that specific underlying context or SA. The affiliation exposure model (AEM) was used to understand if the affiliation to common SA categories (ie, peers who share similar contexts) within a specific CT is associated with user engagement levels in the ADA community. Affiliation exposure measures the percentage of events in the community, where users coparticipate with other users while embracing a specific behavior [34]. This allows characterization of the role of context-specific social influence patterns underlying peer-to-peer communication in digital communities and simultaneously evaluates the extent to which “membership or affiliation” in a specific SA category is associated with user engagement levels.

In this context, we used the network exposure model [34-37] that assumes that social influence occurs when community users are exposed to a specific behavior by their social network contacts. The 2-mode affiliation networks represented a user (row)-by-SA (column) matrix, where each cell entry recorded the number of times a particular SA (*k*) was expressed by the user (*n*; ie, n × k 2-mode valued matrix) in a given CT. This network was binarized using the median of the counts of SA expressed by all community users in a given theme as a threshold and used for further analysis (*A_ij_*)=1or 0 for *i*=1, … , 529, and *k*=1, … ,8. By multiplying this dichotomized 2-mode affiliation matrix (*A_ij_*) with its transpose (*A_ij’_*), the resulting coaffiliation matrix *C* (=*A*_*ij*_*A*_*ij*_) is a symmetric matrix where off-diagonal entries represent the pair of user’s coexpression of SAs during peer conversations. The diagonal entries represent the number of SAs expressed by a specific ADA community user (diagonal vector of *C_ij_*).

The computation of affiliation exposure uses the coaffiliation matrix (*C_ij_*) and multiplies *C_ij_* by each user’s attribute *y_j_* (ie, engagement level, which corresponds to the posting frequency of the ADA users). In this scenario, given that *y_j_* represents a continuous variable, affiliation exposure measures the mean *y* value of all ADA users with whom the ADA user is affiliated through the expression of the same SAs weighted by the shared SAs. The diagonal values of *C_ij_; i=j* were not included in this computation but are included as a control variable for later regression analysis to alleviate the potential underestimation of autocorrelation parameter estimates [34]. The formula used to compute 2-mode affiliation exposure is as follows:


$$\underset{\_}{F}=\frac{\sum_{j=1}C_{ij}\;y_{j}}{\sum_{j=1}C_{ij}}\;for\;i,j=1,\ldots ,N\;i\neq j$$

where *F* is the affiliation exposure vector, *C_ij_* is the coaffiliation matrix that represents a symmetric matrix of community users (user-by-user) with every off-diagonal cell entry recording the number of SAs shared between a pair of ADA users in their peer conversations, and *y_j_* is a vector of user’s behavioral attribute (user’s posting frequency). In this work, affiliation exposure measures the percentage of SAs that ADA users coexpress while engaging with other community users in a given CT. To account for network autocorrelation, we used the 2-mode version of the network autocorrelation model, which is defined as:


y=ρWy+Xβ+γD+ϵfor ϵ∼n(0,σ2I)

where *y* is the vector of the user’s behavioral attribute (user’s posting frequency), *Wy* equivalent to affiliation exposure term F with *W* being (n×n) coexpression matrix *C*, *X*(*n*×*h*) is a matrix of values for the *n* community users on *h* independent variables with unit row vector for the intercept term, *β* (n × h) is a vector of regression coefficients, *ρ* is a scalar estimate of autocorrelation parameter, *D* represents the number of SAs expressed by each community user, and *γ* is the corresponding parameter. The covariates were the number of SAs each user expressed (diagonal vector of *C_ij_*), medication status, diagnosis status, and lifestyle status (*X*s). We used the lnam function from the statnet library in R (R Foundation for Statistical Computing), open-source statistical analysis software for this purpose [38].

## Results

### Characterization of Content and Context Exchanged in Social Ties

#### Qualitative Analysis

Regarding the thematic interests of the ADA community users, social support (1128/1501, 75.1%) was the most communicated theme among users. Teachable moments (357/1501, 23.8%) was the second most prevalent theme among ADA community users, using which the users described how positive behavior changes impacted their blood glucose levels. The medication-related conversations centered around insulin, Lantus, metformin, etc, were quite prevalent (pharmacotherapy: 310/1501, 20.7%). Anxiety issues or the inability to manage blood glucose numbers within the desired range were the most commonly expressed obstacles among ADA community users (obstacles: 262/1501, 17.5%). ADA community users shared patient-reported outcomes (232/1501, 15.5%), for example, the impact of β-blockers on blood glucose readings (Multimedia Appendix 2).

For BCTs, feedback and monitoring (659/1501, 43.9%) was the most frequently used among the community users, followed by social support (565/1501, 37.6%), shaping knowledge (518/1501, 34.5%), antecedents (420/1501, 28%), regulation (323/1501, 21.5%), natural consequences (294/1501, 19.6%), goals and planning (246/1501, 16.4%), and comparison of outcomes (185/1501, 12.3%). Community users provided feedback to one another regarding their self-management behaviors toward diabetes. Users also provided support to one another through emotional support or practical guidance. DHC users guide their peers through information on how behavior can be changed or how to restructure or organize physical or social environments to support positive behavior changes. Discussions on regulating positive behavior through medication options such as insulin and metformin were also present. The community users provided examples of social, emotional, and health consequences of changing their behaviors.

Assertion SA (845/1501, 56.3%) was the most prevalent SA embedded within the ADA messages, such as “consider blurry vision as a sign of high blood sugar” or “diet and exercise are the primary tools of defense against diabetes.” There was also a high prevalence of statement SA (555/1501, 37%) highlighting health-related practices of community users, such as “since my diagnosis I have cut down carbs, started exercising and taking metformin with the goal of keeping A1C values close to normal.” Directive SA (392/1501, 26.1%) highlighting the presence of peer guidance within the community was also prevalent, such as “follow up with your primary care physician to get the medications checked” or “check your blood glucose values at least before every meal in the beginning.” Many community ADA users seeking guidance from their peers posted their queries or questions (304/1501, 20.3%) in the forums. Stance SA (260/1501, 17.3%) in the form of “I agree, meds are a source of consternation” or “I disagree with your point” was also prevalent in ADA peer interactions.

#### Automated Text Analysis

For the classification of CTs, the performance of BERT (ADA-trained) and BERT-base was comparable for all the categories. For progress CT, BERT (ADA-trained) had a higher *F*_1_-score compared to BERT-base, and for obstacles CT, BERT-base had a higher *F*_1_-score compared to BERT (ADA-trained; Table 2). RNNs and CNNs performed comparably to BERT models for determining social support and patient-reported outcomes CTs. The average performance of RNNs and CNNs was comparable, while the average performance of BERT (ADA-trained) and BERT-base was the same. BERT (ADA-trained) outperformed all other models when predicting community-specific pharmacotherapy and progress CTs within ADA peer interactions. It could be because further pretraining on the ADA corpus helped the model to understand the context of words that pertain to medication uses, such as sugar, swings, insulin, and metformin, as well as understand the context of how these community users report their behavioral progress in terms of A_1c_ values over time, etc.

**Table 2.** Category-wise *F*_1_-scores of deep learning models for classification of communication attributes in the American Diabetes Association (ADA) dataset.

Category		RNN^a^	LSTM^b^	BiLSTM^c^	GRU^d^	CNN^e^	BERT^f^-base	BERT (ADA-trained)
**Communication themes**								
	Social support	0.91	0.91	0.88	0.91	0.91	0.91	0.91
	Readiness regulators	0.70	0.76	0.79	0.72	0.78	0.81	0.80
	Pharmacotherapy	0.62	0.67	0.53	0.66	0.68	0.79	0.78
	Obstacles	0.71	0.65	0.69	0.68	0.74	0.75	0.73
	Patient-reported outcomes	0.81	0.81	0.82	0.79	0.79	0.81	0.81
	Progress	0.62	0.69	0.68	0.64	0.56	0.74	0.76
	Average performance (SD)	0.73 (0.11)	0.75 (0.10)	0.73 (0.13)	0.73 (0.10)	0.74 (0.12)	0.80 (0.06)	0.80 (0.06)
**Behavior change techniques**								
	Feedback and monitoring	0.66	0.66	0.59	0.64	0.71	0.72	0.72
	Social support	0.59	0.61	0.55	0.65	0.63	0.71	0.71
	Shaping knowledge	0.60	0.64	0.71	0.66	0.67	0.75	0.78
	Antecedents	0.63	0.68	0.68	0.67	0.70	0.73	0.71
	Regulation	0.66	0.67	0.81	0.62	0.76	0.81	0.86
	Natural consequences	0.68	0.70	0.73	0.72	0.76	0.71	0.74
	Goals and planning	0.78	0.73	0.78	0.76	0.79	0.79	0.79
	Comparison of outcomes	0.57	0.67	0.67	0.58	0.67	0.73	0.76
	Average performance (SD)	0.65 (0.07)	0.67 (0.04)	0.69 (0.09)	0.66 (0.06)	0.71 (0.05)	0.74 (0.04)	0.76 (0.05)
**Speech acts**								
	Assertion	0.71	0.70	0.73	0.68	0.70	0.74	0.76
	Statement	0.49	0.53	0.54	0.47	0.60	0.69	0.71
	Directive	0.38	0.51	0.54	0.49	0.51	0.62	0.67
	Question	0.27	0.45	0.45	0.53	0.54	0.72	0.75
	Emotion	0.62	0.60	0.65	0.68	0.63	0.63	0.72
	Stance	0.53	0.60	0.64	0.56	0.58	0.67	0.71
	Declarative	0.69	0.70	0.71	0.59	0.72	0.67	0.76
	Expressive	0.67	0.68	0.63	0.62	0.68	0.71	0.75
	Average performance (SD)	0.55 (0.16)	0.60 (0.09)	0.61 (0.09)	0.58 (0.08)	0.62 (0.08)	0.68 (0.04)	0.73 (0.03)

a RNN: recurrent neural network.

b LSTM: long short-term memory.

c BiLSTM: bidirectional long-short-term memory.

d GRU: gated recurrent unit.

e CNN: convolutional neural network.

f BERT: Bidirectional Encoder Representations from Transformers.

For BCT classification, BERT (ADA-trained) was better than all other models for classifying various BCT categories, except for the antecedents and natural consequences, for which the BERT-base and CNN had higher predictive performance, respectively. However, the average performance of BERT (ADA-trained) was higher than all other models. The BERT-base model’s performance was comparable to that of BERT (ADA-trained) in predicting feedback and monitoring, social support, and goals and planning BCTs. The BERT-based model’s average performance was comparable to that of BERT (ADA-trained) in classifying various BCT categories.

In the case of SAs, BERT (ADA-trained) achieved the highest *F*_1_-scores for all the categories, ranging from 0.67 to 0.76 (Table 2). The average performance of the model was much higher than that of the other models—BERT-base, CNNs, and RNNs. The *F*_1_-score was lowest for identifying directive SA in the ADA dataset, while assertion, declarative, question, and expressive had the highest *F*_1_-scores (0.76, 0.76, 0.75, and 0.75, respectively).

### Characterization of Individual Behaviors: Qualitative Analysis

We extracted the behavior persona for 529 (~22.3%) ADA community users (from 2374 community users) who had provided their self-reported behavior signatures. The distribution of different statuses is provided in Table 3; as can be seen, most of the users interacting within the ADA forum used oral medications (237/529, 44.8%), had a long history of diabetes (428/529, 80.9%), and did not provide any information about lifestyle changes (378/529, 71.5%).

**Table 3.** User-level behavior persona extracted from the American Diabetes Association dataset.

		Users (n=529), n (%)
**Medication profile**		
	Oral only	237 (44.8)
	Injectable only	63 (11.9)
	Both (oral+injectable)	77 (14.6)
	No medications	52 (9.8)
	No information	102 (19.3)
**Diagnosis profile**		
	Pre-existing diabetes	428 (80.9)
	Newly diagnosed	4 (0.8)
	No information	99 (18.7)
**Lifestyle profile**		
	Yes	153 (28.9)
	No	378 (71.5)

### Characterization of Social Ties

#### Overview

**Figure 2.**
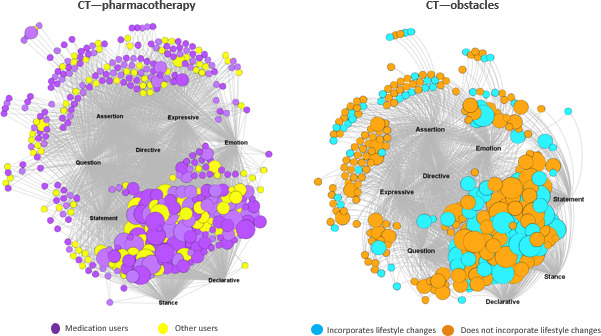
Two-mode affiliation networks for American Diabetes Association community users. CT: communication theme.

For illustration purposes, Figure 2 presents the users-by-SA affiliation networks for ADA community users for the 2 CTs—pharmacotherapy and obstacles. In the pharmacotherapy CT–based network, the purple nodes represent the medication users, and the yellow nodes represent the other users. In the obstacles CT–based network, the blue color nodes represent the users who incorporate lifestyle changes, and the orange nodes represent users who did not incorporate lifestyle changes. In both networks, the size of the nodes represents the engagement of the users, where the large-sized nodes represent the power engagement users, medium-sized ones represent the sustained engagement users, and small-sized nodes represent the infrequent engagement users. The different SA categories are represented by their labels, and the affiliation ties represent the SAs the users expressed in their communication using the 2 CTs. These data represent all users’ communications from 2012 to 2021, in which the ADA users expressed the 2 CTs given. As seen in the figure, stance and declarative are popular SAs among power engagement users in the pharmacotherapy CT–based network.

#### Affiliation Exposure Model

The overall ADA dataset used for AEM spanned from 2014 to 2021, consisting of 56,993 messages organized into 7232 unique topics posted by 529 community users with self-reported signatures. The distribution of messages by themes is provided in Table 4.

**Table 4.** Theme-specific affiliation exposure model dataset characteristics.

Communication themes	Messages (n=56,993), n (%)	Topics (n=7232), n (%)	Users (n=529), n (%)
Social support	56,952 (99.9)	7232 (100)	529 (100)
Readiness regulators	40,233 (70.6)	6726 (93)	505 (95.5)
Pharmacotherapy	20,722 (36.4)	4333 (59.9)	471 (89)
Obstacles	8204 (14.4)	2635 (36.4)	360 (68.1)
Patient-reported outcomes	19,230 (33.7)	3033 (41.9)	391 (73.9)
Progress	18,205 (31.9)	2869 (39.7)	378 (71.5)

The effect of affiliation exposure on user engagement was statistically significant for all CTs (ie, social support, readiness regulators, pharmacotherapy, obstacles, patient-reported outcomes, and progress; communication content). The autocorrelation parameter estimates indicated a positive association between exposure to community users through interactive communication style SAs and user engagement. Specifically, community users affiliating with interactive turn-taking communication styles, such as questions, emotions, or expressive statements, were positively linked to higher engagement levels among ADA community users. For example, when a user with a question about morning glucose levels (communication context) interacts with others sharing a similar DSM context within a readiness-regulators–specific network, they are more likely to remain engaged in the digital community. This engagement is reflected in their posting frequency. On the other hand, exposure to community users affiliating with push-in communication style SAs, such as assertions, declaratives, directives, stances, or statements (communication context), was negatively associated with user engagement in the community (Table 5).

The pre-existing users with T2D were more likely to stay engaged in the community when they expressed patient-reported outcomes and progress CTs (communication content) using interactive communication style SAs (questions, emotion, or expressive; communication context). The number of common SAs as manifested in the interactions exchanged between ADA users were significant across all CTs. It indicated that the more SAs a user expressed through peer interactions within the community, the more likely the user would remain engaged with the community for self-managing diabetes-related behaviors (Table 5).

**Table 5.** Affiliation exposure among American Diabetes Association users derived from the network autocorrelation model.

Type of CTs^a^ and type of SAs^b^ (communication styles)		Affiliation exposure, b (SE)	Medication status, b (SE)	Diagnosis status, b (SE)	Lifestyle status, b (SE)	SAs affiliated, b (SE)
**Social support (n=529)**						
	Push-in CS^c^	−0.012^d^ (0.004)	−0.790 (1.415)	1.682 (1.516)	−1.500 (2.068)	0.758^d^ (0.002)
	Interactive turn-taking CS	0.068^d^ (0.006)	−0.758 (2.560)	0.885 (2.747)	1.787 (3.753)	0.929^d^ (0.004)
**Readiness regulators (n=505)**						
	Push-in CS	−0.023^d^ (0.003)	−0.546 (0.812)	0.837 (0.865)	−1.222 (1.167)	0.731^d^ (0.001)
	Interactive turn-taking CS	0.067^d^ (0.006)	−0.825 (1.884)	0.660 (2.008)	3.200 (2.707)	0.885^d^ (0.004)
**Pharmacotherapy (n=471)**						
	Push-in CS	−0.012^e^ (0.004)	0.160 (0.474)	0.500 (0.504)	−1.003 (0.688)	0.690^d^ (0.002)
	Interactive turn-taking CS	0.074^d^ (0.007)	−0.391 (1.000)	0.212 (1.064)	1.080 (1.452)	0.871^d^ (0.005)
**Obstacles (n=360)**						
	Push-in CS	−0.017^d^ (0.003)	−0.209 (0.147)	0.049 (0.162)	0.102 (0.222)	0.735^d^ (0.002)
	Interactive turn-taking CS	0.070^d^ (0.008)	−0.265 (0.441)	0.663 (0.488)	0.164 (0.668)	0.839^d^ (0.006)
**Patient-reported outcomes (n=391)**						
	Push-in CS	−0.008^e^ (0.003)	−0.246 (0.312)	0.516 (0.340)	−0.313 (0.453)	0.707^d^ (0.001)
	Interactive turn-taking CS	0.080^d^ (0.006)	−0.590 (0.754)	1.854^f^ (0.823)	1.208 (1.094)	0.821^d^ (0.004)
**Progress (n=378)**						
	Push-in CS	−0.009^d^ (0.003)	−0.258 (0.313)	0.512 (0.343)	−0.279 (0.456)	0.708^d^ (0.002)
	Interactive turn-taking CS	0.082^d^ (0.006)	−0.421 (0.752)	1.944^f^ (0.823)	0.822 (1.092)	0.820^d^ (0.004)

a CT: communication theme.

b SA: speech act.

c CS: communication style.

d *P*<.001.

e *P*<.01.

f *P*<.05.

## Discussion

### Principal Findings

#### Overview

Studies on social diffusion research underscore social relationships’ role in the adoption and spread of behaviors [39]. Ideological proximity increases the likelihood of individuals becoming friends and influences the dynamic of social interactions [40-43]. Characterizing the communication content and context embedded in these social exchanges helps capture the proximity of such ideas. Communication attributes captured via CTs, BCTs, and SAs, along with the structure of social ties in a DHC, can provide us with insights into mechanisms of how communication events lead to specific social actions. One study showed that highly engaged individuals with the diabetes digital community achieve better health outcomes, such as improved glycemic levels, than those who do not engage with such digital platforms [44].

In this paper, we described our attempts to adapt the existing advances in natural language processing techniques and social network modeling approaches to incorporate communication-level attributes (content and context) and individual-level attributes to understand the social influence mechanisms that drive user DSM behaviors from large-scale social media datasets. This study takes an empirically grounded approach to derive communication content- and context-driven network patterns of behavior change that can be translated into the design of adaptive BCIs. The 2-mode affiliation networks allowed us to visualize distinctive patterns of clustering within CT- and BCT-specific networks. The community users in these affiliation networks are interconnected by different SAs, with certain SAs being more popular than others as per user’s engagement status, and it also varies by various kinds of CTs or BCTs. Another study used affiliation networks to study the impact of affiliation on alcohol use behaviors among adolescents [45]. Young et al [46] investigated how affiliation to certain digital groups within a social network can influence sexual behaviors. Overall, the results from content-sensitive and context-aware SNA conducted in our work reveal multiple significant patterns of expression of specific content and context that can influence users’ DSM behaviors.

#### Implications for Design of Digital DSM Interventions

The results from this study indicate that capturing various communication attributes from digital peer conversations can help us understand users’ implicit needs and how providing users with their requirements can positively impact their DSM behaviors. For example, users expressing themselves with specific communication attributes (eg, interactive turn-taking SAs) can form better connections with other community users, which was shown to improve engagement in DSM behaviors [47]. Our results from AEMs show that specific patterns of content and context can exert social influence—for example, ADA community users affiliating with peers who express with interactive turn-taking communication style SAs in the form of question, expressive, or emotion tend to stay engaged in the community. In another study, the AEM was used to understand how affiliation-based peer influence affects alcohol use behaviors in adolescents [48]. Previous studies have shown how user engagement in social media can influence their health-related outcomes [49,50]. Social network interventions using the use of such networks have already been proposed by researchers in the domain of HIV prevention [51] and tackling COVID-19 misinformation spread [52]. The findings from this study suggest new directions in developing network interventions that focus on incorporating communication attributes that are personalized to individuals’ latent needs. For example, an intervention in the form of an artificial intelligence Bot Moderator can recommend connections to make structural changes to the existing networks, such as connecting users with similar contexts, for example, a community user asking questions about pharmacological support can be recommended to communicate with other users who have similar questions.

### Limitations

First, in the qualitative analysis, the relatively small sample size was selected for manual annotation, which may have resulted in inaccurate representations of the overall prevalence of different communication attributes. However, the sample of 1501 messages using qualitative research methods was appropriate for the research objectives. For this research, we extracted messages about topics related to T2D, and the extractions were done in 2018 and 2021. While there was a reduction in the number of messages in our dataset between 2018 and 2021, several external factors must be considered, notably the community’s transition to a new technology platform and the impact of the COVID-19 pandemic. Research during the pandemic has shown that DSM behaviors were significantly impacted, with many individuals experiencing both positive (adopting healthier eating habits) and negative (decreased physical activity) changes in their management routines due to social isolation, stress, and disruptions in health care access [53,54]. It aligns with what may have occurred within our study community, as they faced the dual challenge of adapting to a new platform and managing the broader societal disruptions caused by the pandemic. Despite these challenges, the dataset remains highly relevant to understanding DSM, as peer interactions are a cornerstone of diabetes self-care. The insights from this dataset contribute to a broader understanding of how peer support can enhance patient engagement in DSM. Thus, while the reduction in message volume is a limitation, the remaining interactions continue to provide valuable insights into the adaptation and resilience of individuals managing diabetes in digital social environments. Second, we only considered some categories of BCTs and SAs for automated text analysis, given the imbalanced nature of the manually annotated dataset. In addition, while applying the finalized model to the unlabeled dataset, we used the threshold values for assigning a particular category of CTs, BCTs, or SAs to the peer messages, which reduced the total number of labeled messages, which might have resulted in missed network ties during our retrospective and SNA. Finally, the AEM analysis was based on the cross-sectional affiliation data obtained from the ADA dataset, which limits our understanding of the potential causality of SA affiliation and dynamic patterns of SA affiliation in various CT-based social networks. Despite this limitation, this work offers empirical insights into users’ affiliation to SAs using certain themes or theoretical constructs. Another critical limitation of this study is the potential for bias arising from affiliation exposure, particularly selection bias, autocorrelation bias, and the challenge of distinguishing between causality and correlation [34]. Selection bias may occur if the dataset overrepresents certain affiliations, leading to results that are not fully generalizable. Our methods attempt to address this by ensuring random harvesting of digital interactions. However, our data are limited to individuals participating in these networks. Future works should attempt to include mixed methods recruitment strategies to ensure broader population-level data capture. Autocorrelation bias can inflate behavioral similarities within networks, making it appear that behaviors spread more widely due to social connections rather than inherent trends [34]. Although our AEM helps mitigate these biases by segregating peer and group influences, the difficulty in separating correlation from causality remains. While individuals within certain affiliations may exhibit similar behaviors, it is often unclear whether these behaviors are driven by the affiliation itself or by pre-existing characteristics that led individuals to join those groups. Future research should aim to diversify affiliations in the dataset and incorporate longitudinal data to address these biases better and distinguish between correlation and causality.

We extracted behavioral profiles for only a subset of the community users with self-reported behavior persona; thus, such behavior profiles may not represent the entire community user population. Moreover, this analysis does not consider sociodemographic and cultural factors, which can also result in differences in the expression of various communication attributes. Future work should focus on complementing the current efforts by biobehavioral sensing using commercial wearables (such as continuous glucose monitors), collaborating with community partners, and using data obtained from multiple communities for each application domain as has been used by other studies [55]. Such insights will help us understand users’ needs and triggers surrounding certain behavioral events (such as fluctuations in blood glucose values) so that the interventions can be customized for that specific behavioral stage of change.

### Conclusions

Ubiquitous internet connectivity has led to the onset of digital health platforms where more and more individuals are engaging with their peers to manage their health-related conditions. Our study demonstrates that real-time digital interactions effectively capture the complexities of DSM-related behaviors and reveal how self-expression within specific contexts influences engagement with digital peers, ultimately affecting DSM. A theory-driven, large-scale analysis of such datasets can provide valuable insights into the underlying processes of DSM, informing the design of highly effective BCIs.

## Supplementary material

10.2196/60109 Multimedia Appendix 1 Example of affiliation ties in the American Diabetes Association community.

10.2196/60109 Multimedia Appendix 2 Distribution of (A) communication themes, (B) behavior change techniques, and (C) speech acts in the American Diabetes Association community.

## Abbreviations

ADA: American Diabetes Association
AEM: affiliation exposure model
BCI: behavior change intervention
BCT: behavior change technique
BERT: Bidirectional Encoder Representations from Transformers
CNN: convolutional neural network
CT: communication theme
DHC: digital health community
DSM: diabetes self-management
RNN: recurrent neural network
SA: speech act
SNA: social network analysis
T2D: type 2 diabetes

## References

[1] (2022). National Diabetes Statistics Report | Diabetes. Centers for Disease Control and Prevention.

[2] Magliano DJ, Boyko EJ (2021). IDF Diabetes Atlas.

[3] Haas L, Maryniuk M, Beck J (2012). National standards for diabetes self-management education and support. Diabetes Educ.

[4] Glanz K, Rimer BK, Viswanath K (2008). Health Behavior and Health Education Theory, Research, and Practice.

[5] Dietz W, Brownson R, Douglas C (2016). Improving Physical Activity and Nutrition and Reducing Tobacco Use and Obesity to Prevent Chronic Disease. Discussion Paper, Vital Directions for Health and Health Care Series.

[6] Christakis NA, Fowler JH (2008). The collective dynamics of smoking in a large social network. N Engl J Med.

[7] Christakis NA, Fowler JH (2007). The spread of obesity in a large social network over 32 years. N Engl J Med.

[8] Marsch LA (2021). Digital health data-driven approaches to understand human behavior. Neuropsychopharmacology.

[9] Centola D (2013). Social media and the science of health behavior. Circulation.

[10] Fisher J, Clayton M (2012). Who gives a tweet: assessing patients’ interest in the use of social media for health care. Worldviews Evid Based Nurs.

[11] Chou WYS, Prestin A, Lyons C, Wen K yi (2013). Web 2.0 for health promotion: reviewing the current evidence. Am J Public Health.

[12] Chou WYS, Hunt YM, Beckjord EB, Moser RP, Hesse BW (2009). Social media use in the United States: implications for health communication. J Med Internet Res.

[13] Oser TK, Oser SM, Parascando JA (2020). Social media in the diabetes community: a novel way to assess psychosocial needs in people with diabetes and their caregivers. Curr Diab Rep.

[14] Myneni S, Fujimoto K, Cohen T, Patel VL, Arocha JF, Ancker JS (2017). Cognitive Informatics in Health and Biomedicine: Understanding and Modeling Health Behaviors.

[15] Singh T, Roberts K, Cohen T (2020). Social Media as a Research Tool (SMaaRT) for risky behavior analytics: methodological review. JMIR Public Health Surveill.

[16] Da Moura Semedo C, Bath PA, Zhang Z (2023). Social support in a diabetes online community: mixed methods content analysis. JMIR Diabetes.

[17] Yao Z, Zhang B, Ni Z, Ma F (2022). What users seek and share in online diabetes communities: examining similarities and differences in expressions and themes. AJIM.

[18] Jiang S, Liu X, Chi X (2022). Effect of writing style on social support in online health communities: a theoretical linguistic analysis framework. Inf Manag.

[19] Austin JL (1962). How to Do Things with Words.

[20] Searle JR (1969). Speech Acts: An Essay in the Philosophy of Language.

[21] (2023). Research, education, advocacy. American Diabetes Association.

[22] Myneni S, Cobb N, Cohen T (2016). In pursuit of theoretical ground in behavior change support systems: analysis of peer-to-peer communication in a health-related online community. J Med Internet Res.

[23] Michie S, Richardson M, Johnston M (2013). The behavior change technique taxonomy (v1) of 93 hierarchically clustered techniques: building an international consensus for the reporting of behavior change interventions. Ann Behav Med.

[24] Myneni S, Lewis B, Singh T (2020). Diabetes self-management in the age of social media: large-scale analysis of peer interactions using semiautomated methods. JMIR Med Inform.

[25] Singh T, Olivares S, Cohen T (2021). Pragmatics to reveal intent in social media peer interactions: mixed methods study. J Med Internet Res.

[26] Alpaydin E, Kaynak C (1988). Cascading classifiers. Kyber.

[27] Singh VK, Shrivastava U, Bouayad L, Padmanabhan B, Ialynytchev A, Schultz SK (2018). Machine learning for psychiatric patient triaging: an investigation of cascading classifiers. J Am Med Inform Assoc.

[28] Kingma DP, Ba J Adam: a method for stochastic optimization.

[29] Chollet F (2018). Deep Learning with Python.

[30] Paszke A, Gross S, Chintala S (2017). Automatic differentiation in PyTorch. OpenReview.

[31] Singh T, Roberts K, Cohen T, Cobb N, Franklin A, Myneni S (2023). Discerning conversational context in online health communities for personalized digital behavior change solutions using Pragmatics to Reveal Intent in Social Media (PRISM) framework. J Biomed Inform.

[32] Powers MA, Bardsley J, Cypress M (2015). Diabetes self-management education and support in type 2 diabetes: a joint position statement of the American Diabetes Association, the American Association of Diabetes Educators, and the Academy of Nutrition and Dietetics. Diabetes Care.

[33] Bastian M, Heymann S, Jacomy M Gephi: an open source software for exploring and manipulating networks. https://www.aaai.org/ocs/index.php/ICWSM/09/paper/view/154.

[34] Fujimoto K, Chou CP, Valente TW (2011). The network autocorrelation model using two-mode data: affiliation exposure and potential bias in the autocorrelation parameter. Soc Networks.

[35] Burt RS (1987). Social contagion and innovation: cohesion versus structural equivalence. Am J Sociol.

[36] Valente TW (1995). Network Models of the Diffusion of Innovations.

[37] Leenders RThAJ (2002). Modeling social influence through network autocorrelation: constructing the weight matrix. Soc Networks.

[38] Butts CT (2008). Social network analysis with SNA. J Stat Soft.

[39] Rogers EM (1995). Diffusion of Innovations.

[40] Fujimoto K, Valente TW (2012). Social network influences on adolescent substance use: disentangling structural equivalence from cohesion. Soc Sci Med.

[41] Fujimoto K, Williams ML, Ross MW (2013). Venue-based affiliation networks and HIV risk-taking behavior among male sex workers. Sex Transm Dis.

[42] Valente TW, Gallaher P, Mouttapa M (2004). Using social networks to understand and prevent substance use: a transdisciplinary perspective. Subst Use Misuse.

[43] Valente TW, Hoffman BR, Ritt-Olson A, Lichtman K, Johnson CA (2003). Effects of a social-network method for group assignment strategies on peer-led tobacco prevention programs in schools. Am J Public Health.

[44] Litchman ML, Edelman LS, Donaldson GW (2018). Effect of diabetes online community engagement on health indicators: cross-sectional study. JMIR Diabetes.

[45] Leung RK, Toumbourou JW, Hemphill SA (2014). The effect of peer influence and selection processes on adolescent alcohol use: a systematic review of longitudinal studies. Health Psychol Rev.

[46] Young LE, Fujimoto K, Schneider JA (2018). HIV prevention and sex behaviors as organizing mechanisms in a Facebook group affiliation network among young black men who have sex with men. AIDS Behav.

[47] Moulaei K, Dinari Z, Dinari F, Jahani Y, Bahaadinbeigy K (2022). The role of social networks in diabetes self-care: a cross-sectional study. Health Sci Rep.

[48] Fujimoto K, Valente TW (2013). Alcohol peer influence of participating in organized school activities: a network approach. Health Psychol.

[49] Chen J, Wang Y (2021). Social media use for health purposes. Syst Rev J Med Internet Res.

[50] Ghahramani A, de Courten M, Prokofieva M (2022). The potential of social media in health promotion beyond creating awareness: an integrative review. BMC Public Health.

[51] Pagkas-Bather J, Young LE, Chen YT, Schneider JA (2020). Social network interventions for HIV transmission elimination. Curr HIV/AIDS Rep.

[52] Young LE, Sidnam-Mauch E, Twyman M (2021). Disrupting the COVID-19 misinfodemic with network interventions: network solutions for network problems. Am J Public Health.

[53] Zupa MF, Perez S, Palmisano G (2022). Changes in self-management during the COVID-19 pandemic among adults with type 2 diabetes at a federally qualified health center. J Immigr Minor Health.

[54] Sauchelli S, Bradley J, England C, Searle A, Whitmarsh A (2021). Exploring support needs of people living with diabetes during the coronavirus COVID-19 pandemic: insights from a UK survey. BMJ Open Diabetes Res Care.

[55] Frey E, Bonfiglioli C, Frawley J (2021). Self-management: diabetes care is getting social. BMJ.

