# Correction: Toward Personalized Digital Experiences to Promote Diabetes Self-Management: Mixed Methods Social Computing Approach

**DOI:** 10.2196/75497

**Published:** 2025-04-18

**Authors:** Tavleen Singh, Kirk Roberts, Kayo Fujimoto, Jing Wang, Constance Johnson, Sahiti Myneni

**Affiliations:** 1 McWilliams School of Biomedical Informatics, The University of Texas Health Science Center, 7000 Fannin, Houston, TX, 77030, United States, 1 713 500 3900; 2 School of Public Health, The University of Texas Health Science Center, Houston, TX, United States; 3 College of Nursing, Florida State University, Tallahassee, FL, United States; 4 Cizik School of Nursing, The University of Texas Health Science Center, Houston, TX, United States

**Keywords:** digital health communities, diabetes self-management, behavior change, affiliation exposure, social networks, deep learning

In “Toward Personalized Digital Experiences to Promote Diabetes Self-Management: Mixed Methods Social Computing Approach” (JMIR Diabetes 2025;10:e60109) the authors made one addition.

In the Acknowledgements, the following line was added:

This work was supported by NIH award numbers 1R01LM012974-01A1 & 1R01AG089193-01.

The correction will appear in the online version of the paper on the JMIR Publications website, together with the publication of this correction notice. Because this was made after submission to PubMed, PubMed Central, and other full-text repositories, the corrected article has also been resubmitted to those repositories.

