# Supplementary figures and images for: Toward Personalized Digital Experiences to Promote Diabetes Self-Management: Mixed Methods Social Computing Approach
Source: JMIR Diabetes. 2025 Jan 7;10:e60109. doi: 10.2196/60109 (PMC11731698; doi:10.2196/60109)

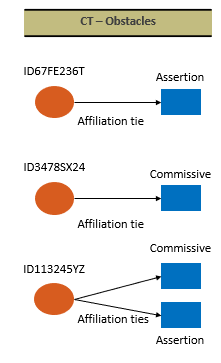

Supplement: Multimedia Appendix 1 [file diabetes-v10-e60109-s001.png]

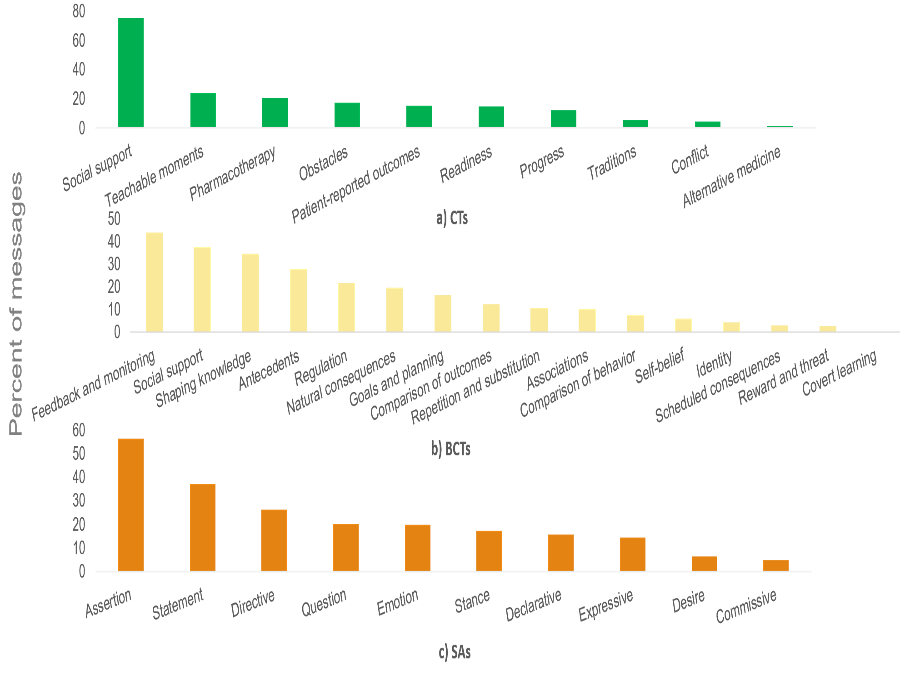

Supplement: Multimedia Appendix 2 [file diabetes-v10-e60109-s002.png]
